# Editorial: Advances in the Diagnosis and Treatment of Skull Base Tumours

**DOI:** 10.3389/fonc.2022.887595

**Published:** 2022-05-25

**Authors:** Eoin F. Cleere, Aviram Mizrachi, Marc A. Cohen, James Paul O’Neill

**Affiliations:** ^1^ Department of Otolaryngology, Royal College of Surgeons in Ireland, Dublin, Ireland; ^2^ Department of Head and Neck Surgery, Beaumont Hospital, Dublin, Ireland; ^3^ Department of Head and Neck Surgery, Rabin Medical Centre, Petah-Tikvah, Israel; ^4^ Department of Head and Neck surgery, Memorial Sloan Kettering Cancer Center, New York, NY, United States

**Keywords:** Skull-base, neoplasia, surgery, radiotherapy, cancer

In this Frontiers Research Topic we sought to highlight some of the most recent advances regarding the diagnosis and treatment of tumours affecting the skull base. The anatomy of the skull base region is complex with numerous critical neurovascular structures in close proximity. Thus, the management of tumours in this region poses a unique challenge for surgeons in order to not only achieve good oncologic outcomes but also to minimize treatment associated morbidity (1, 2). As a result of this anatomical complexity it has been necessary to develop innovative surgical approaches to these tumours. One such advancement in this regard is the introduction of the endoscopic endonasal approach to anterior skull base tumours, in particular for pituitary tumours (3). Van Gerven et al. detailed their initial 10 – year experience with the introduction of this approach within their institution. They retrospectively analysed 369 patients (87.3%; 322/369 pituitary adenomas) with sellar and suprasellar tumours managed in this way. They demonstrated that operative time decreased as surgeon familiarity with the technique increased and favourable outcomes with the endoscopic endonasal approach were observed with a recurrence rate of 20.0% following pituitary adenoma resection. Overall 7.3% (27/369) of their patients suffered a cerebrospinal fluid (CSF) leak postoperatively. CSF leaks are a dreaded complication following endoscopic endonasal resection of anterior skull base tumours and reconstructive approaches to reduce the incidence of this complication were the focus of the review article by Hannan et al. The incidence of CSF leaks following the endoscopic endonasal approach were initially seen as the barrier to the widespread incorporation of this approach into surgical practice (4). The introduction of nasoseptal flap (NSF) as part of a multilayer closure has been effective in reducing the incidence of CSF leak in these cases to below 5% in recent times. Hannan et al. also describe their own ‘Dublin technique’ which has resulted in a 1% (1/90) incidence of postoperative CSF leak since its introduction within their institution. They also discuss adjunct methods which may reduce the incidence of postoperative CSF leaks such as the prophylactic use of a lumbar drain. This was the subject of the meta-analysis performed by Guo et al. Their analysis of 8 studies demonstrated that routine lumbar drain use did not significantly reduce the incidence of postoperative CSF leak (OR 0.8; 95% CI 0.37 – 1.74; P=0.57) while routine use of lumbar drain increased the incidence of headache in patients postoperatively.

In tumours affecting the pterygopalatine fossa the maxillary-swing approach (5) is frequently used to access the tumour during surgical resection. However, this approach maintains some inherent limitations such as the close margin at the site of the posterior osteotomy site as well as leaving the surrounding canals and foramina (which may harbour tumour cells) undisturbed. Xie et al. have proposed a novel modification to the classic maxillary-swing approach in order to overcomes some of these limitations. They demonstrated a series of 7 patients with pterygopalatine fossa tumours managed with their modified maxillary-swing approach; achieving en-bloc resection in all 7 cases. One patient (1/7; 14.3%) suffered a locoregional recurrence and no functional morbidity outside of expected facial numbness and epiphora post-operatively was reported.

Despite the major advances in surgical techniques, tumours that affect craniofacial structures still largely require multimodal treatment strategies to achieve local disease control. This was the subject of the review by Konig et al. Their exploration of the literature found that esthenioneuroblastoma and soft tissue sarcomas benefitted from radiotherapy-based adjuvant or neoadjuvant treatment combined with surgery. Sinonasal undifferentiated carcinoma, craniofacial osteosarcoma and neuroendocrine paranasal sinus tumours benefitted from neoadjuvant chemotherapy or adjuvant chemoradiotherapy when combined with surgical resection. On the other hand mucosal melanoma and grade II/III meningiomas appear to be best managed with upfront surgical resection and adjuvant radiotherapy based treatment.

In contrast, radiotherapy-based treatment is utilized as a primary management strategy for nasopharyngeal carcinoma (6). Hua et al. performed an analysis of 1,292 patients with nasopharyngeal carcinoma treated using intensity modulated radiation therapy (IMRT) and concurrent cisplatin. They explored their hypothesis that a prolonged duration of IMRT (IMRT delivered over > 7 weeks) would predispose patients to poor survival outcomes. Patients all received 66 – 70Gy in between 28 – 33 fractions. The prolonged duration of radiotherapy group displayed a significantly worse overall survival (OS) (87.2% v 78.4%; P<0.001) as well as worse distant metastatic free survival, progression free survival and an increased rate of locoregional recurrence. This highlights the necessity of avoiding RT delivery delays in the management of nasopharyngeal carcinoma, a particularly timely finding in the COVID-19 era.

Unfortunately, despite the many advances in the management of skull base tumours many patients still present with advanced disease and an unfavourable prognosis. Komune et al. explored the anatomical factors that impacted survival outcomes in T4 squamous cell carcinoma of the temporal bone. Their retrospective analysis demonstrated that tumour invasion of ossicles, posterior fossa dura or the sigmoid sinus were independent predictors of a reduced 5 year OS. Based with this knowledge they devised a novel 3 factor prognostic classification system for T4 temporal bone squamous cell carcinomas (1. Pterygoid musculature involvement, 2. Ossicular involvement, 3. Posterior fossa dura OR sigmoid sinus involvement). Involvement of an increased number of these structures demonstrated a downward stepwise trend in OS (0 structures involved – 90.9% OS; 1 structure 42.9% OS; 2 structures 25.0% OS; 3 structures – 0.0% OS)

Safi et al. performed a systematic review of the literature to explore the management and outcomes in paediatric patients with esthenioneuroblastoma. Their systematic review of 7 studies and 94 patients suggests that paediatric patients have a tendency to present with advanced disease (69.1%; 65/94 Kadish stage C/D: 20.2%; 19/94 with nodal disease). Paediatric patients also undergo aggressive multimodal therapy with 50% (47/94) of cases receiving triple modality treatment (surgery, radiotherapy and chemotherapy) with the net result of aggressive disease and aggressive therapy being a 5 year OS between 44 – 91% among the included studies.

Finally, this Research Topic was rounded off by a novel lipidomic analysis study by Yu and Wang. They sought to define lipid biomarkers to enable the early diagnosis of laryngeal cancer. Their lipidomic analysis of 29 patients with laryngeal cancer and 36 healthy controls demonstrated that lysophospholipids and phospholipids may serve as potential biomarkers in the early diagnosis of laryngeal cancer.

## Author Contributions

EFC drafted the original manuscript. All authors listed have revised the text and made a substantial, direct, and intellectual contribution to the work and approved it for publication.

## Conflict of Interest

The authors declare that the research was conducted in the absence of any commercial or financial relationships that could be construed as a potential conflict of interest.

## Publisher’s Note

All claims expressed in this article are solely those of the authors and do not necessarily represent those of their affiliated organizations, or those of the publisher, the editors and the reviewers. Any product that may be evaluated in this article, or claim that may be made by its manufacturer, is not guaranteed or endorsed by the publisher.
